# Structural and Functional Changes in Aged Skin Lymphatic Vessels

**DOI:** 10.3389/fragi.2022.864860

**Published:** 2022-04-04

**Authors:** Raghu P. Kataru, Hyeung Ju Park, Jinyeon Shin, Jung Eun Baik, Ananta Sarker, Stav Brown, Babak J. Mehrara

**Affiliations:** The Department of Surgery, Division of Plastic and Reconstructive Surgery, Memorial Sloan Kettering Cancer Center, New York, NY, United States

**Keywords:** dermal lymphatics, age-related lymphatic dysfunction, peri-lymphatic inflammation, lymphatic endothelial apoptosis, decreased LEC VEGFR-3 signaling

## Abstract

Lymphatic structure and function play a critical role in fluid transport, antigen delivery, and immune homeostasis. A dysfunctional lymphatic system is associated with chronic low-grade inflammation of peripheral tissues, poor immune responses, and recurrent infections, which are also hallmarks of aging pathology. Previous studies have shown that aging impairs lymphatic structure and function in a variety of organ systems, including the intestines and central nervous system. However, previous studies are mostly limited to qualitative analysis of lymphatic structural changes and quantification of intestinal collecting vessel contractile function. It is not clear whether decreased lymphatic function contributes to pathological conditions related to aging, nor how it affects the skin immune microenvironment. Further, the effects of aging on skin initial and collecting lymphatic vessels, dendritic cell (DC) migration, cutaneous lymphatic pumping, and VEGFR-3 signaling in lymphatic endothelial cells (LECs) have not been quantitatively analyzed. Here, using fluorescent immunohistochemistry and flow cytometry, we confirm that aging decreases skin initial and collecting lymphatic vessel density. Indocyanine green (ICG) lymphangiography and DC migration assays confirm that aging decreases both fluid pumping and cell migration via lymphatic vessels. At the cellular level, aging causes decreased VEGFR-3 signaling, leading to increased LEC apoptosis and senescence. Finally, we determined that aging causes decreased lymphatic production of chemokines and alters LEC expression of junctional and adhesion molecules. This in turn leads to increased peri-lymphatic inflammation and nitrosative stress that might contribute to aging pathology in a feed-forward manner. Taken together, our study, in addition to quantitatively corroborating previous findings, suggests diverse mechanisms that contribute to lymphatic dysfunction in aging that in turn exacerbate the pathology of aging in a feed-forward manner.

## Introduction

The lymphatic system is an organized, hierarchical vascular network consisting of successively larger vessels that are present in virtually all organ systems and transport tissue fluids, immune cells, macromolecules, antigens, pathogens, and lipids (Oliver et al., 2020; Petrova and Koh, 2020). Lymphatic capillary vessels, also known as initial lymphatics, take up fluid and macromolecules in response to tissue distention. Initial lymphatics drain into pre-collectors and larger collecting vessels that maintain vascular integrity through tight “zipper-like” adherent junctions (Baluk et al., 2007), and pump their contents toward draining lymph nodes through the action of lymphatic muscle cells (LMCs) (von der Weid and Zawieja, 2004; Scallan et al., 2016). In the lymph nodes, antigens and pathogens in lymph are filtered and taken up by antigen-presenting cells that initiate immune responses; the filtered interstitial fluid is eventually returned to the venous system through lymphatico-venous junctions (Randolph et al., 2005; Liao and Padera, 2013).

Clinical and animal studies have shown that aging impairs lymphatic function in the skin, intestines, and central nervous system (CNS) (Akl et al., 2011; Karaman et al., 2015; Ma et al., 2017). Aged mice have decreased lymphatic vessel density, increased lymphatic permeability, and decreased ability to clear bacteria (Zolla et al., 2015). Functional studies have shown that aged rats have decreased lymphatic muscle cell coverage of mesenteric lymphatics, decreased muscle contractile and ion channel protein expression, and decreased collecting lymphatic ejection fraction (Gasheva et al., 2007; Nagai et al., 2011). These findings are consistent with large clinical studies reporting decreased interstitial transport and impaired collecting vessel pumping in healthy geriatric volunteers compared with young controls (Unno et al., 2011).

More recent studies have suggested that age-related lymphatic dysfunction plays a key role in diseases that commonly affect the elderly. For example, mice with age-related decreases in lymphatic clearance of macromolecule and waste products from the CNS have cognitive impairment and amyloid deposition (Da Mesquita et al., 2018). However, how age-associated lymphatic dysfunction affects skin immune and stromal microenvironment is not well known. Because the lymphatic system plays an important role in antigen delivery and induction of immune responses, it is possible that age-related lymphatic dysfunction may also contribute to other pathologic conditions that affect the elderly, including impaired immune responses to tumors or infections, autoimmunity, and arthritis. Thus, understanding the mechanisms that regulate lymphatic dysfunction in aging is an important goal.

Previous studies have suggested that histamine released by mast cells is a negative regulator of lymphatic pumping and contributes to age-related lymphatic dysfunction (Chatterjee and Gashev, 2012; Nizamutdinova et al., 2016; Pal et al., 2017). However, the effects of age-related changes on peri-lymphatic accumulation of other leukocytes remain unknown. This is important because previous studies have shown important roles for T cells and macrophages in regulating lymphatic function and leakiness (Reynolds et al., 2016; Kataru et al., 2019). In addition, while it is clear that aging has significant effects on lymphatic vessel density and function, the cellular and molecular changes that regulate age-related changes in lymphatic endothelial cells (LECs) remain unknown. For example, it is not clear how aging modulates LEC intracellular signaling or if aging increases LEC apoptosis or senescence. Understanding these mechanisms may therefore identify putative treatment options that may mitigate the negative effects of aging on the lymphatic system.

In this study, we performed a comparative analysis of young (2–3-month-old) and aged (8–22-month-old) mouse skin initial and collecting lymphatic vessels encompassing some of the missing aspects from the existing literature. We sought to characterize the effects of aging on structure and function of lymphatic vessels in the skin in terms of density (imaging and flow cytometry), pumping (ICG lymphangiography), dendritic cell migration, and VEGFR-3 signaling in LECs, as well as the effects of LEC changes on the immune microenvironment.

## Materials and Methods

### Mice and Reagents

All experimental protocols were reviewed and approved by the Institutional Animal Care and Use Committee at Memorial Sloan Kettering Cancer Center (MSK). MSK adheres to the National Institutes of Health Guide for the Care and Use of Laboratory Animals and operates in accordance with the Animal Welfare Act. All mice were maintained in a pathogen-free, temperature- and light-controlled environment and provided with normal chow diet and freshwater ad libitum.

Experiments were performed using a combination of male and female mice between 8 and 80 weeks of age. Adult (2- to 3-month-old) C57BL/6J mice (The Jackson Laboratory; Bar Harbor, ME) were included in the young group, while mice aged 18–22 months were considered old. When indicated, anesthesia was induced using isoflurane (Henry Schein Animal Health; Dublin, OH). Respiratory rate and tail pinching were used to monitor the depth of anesthesia. At the conclusion of each experiment, the appropriate animals were euthanized by carbon dioxide asphyxiation as recommended by the American Veterinary Medical Association.

### Fluorescent Immunohistochemistry

Fluorescent immunohistochemical (IHC) staining was performed using standard protocols. Tissues were fixed in 4% paraformaldehyde (Affymetrix, Inc.; San Diego, CA) at 4°C, embedded in Tissue-Tek optimal cutting temperature compound (Sakura Finetek; Torrance, CA) or paraffin, and sectioned at 5–10 µm. All tissue sections were rehydrated prior to staining. Rehydrated paraffin sections were subjected to 15 min of heat-induced (90°C) epitope retrieval in sodium citrate (Sigma-Aldrich) buffer in a water bath. For wholemount staining, ear pinnae were collected, depilated, fixed in 4% PFA for 2 h at room temperature. Fixed ear skins were peeled, cartilage was removed from anterior side, and both anterior and posterior sides were stained by pinning to a Syl Gard gel (Corning; Cat# 24236-10) in a six well plate. Ear skins were washed for 30 min with phosphate-buffered saline (PBS) with 1% Triton X-100 (Sigma-Aldrich). For IHC of sections and wholemounts, non-specific binding was blocked with a solution of 5% donkey or goat serum (Sigma-Aldrich; St. Louis, MO) for 1 h at room temperature. All tissues/sections were then incubated overnight at 4°C with the appropriate primary antibodies (Table 1). Sections or whole-mount tissue preparations were subsequently washed with phosphate-buffered saline (PBS) with 1% Triton X-100 (Sigma-Aldrich) and incubated with corresponding fluorescent-labeled secondary antibody conjugates (AlexaFluor 488, 594, or 647; Life Technologies; Carlsbad, CA) for 5 h followed by 4,6-diamidino-2-phenylindole (DAPI; #D4571, Molecular Probes/Invitrogen; Eugene, OR) for 10 min before mounting with Mowiol (Sigma-Aldrich). Slides stained only with secondary antibodies without primary antibody incubation were used as a negative control.

**TABLE 1:** Antibodies used for immunohistochemistry.

Antigen	Antibody type	Dilution	Catalog number	Company
LYVE-1	Goat polyclonal	1:400	2125-LY	R&D Systems (Minneapolis, MN)
VE-cadherin	Goat polyclonal	1:500	AF1002	R&D Systems
LYVE-1	Rabbit polyclonal	1:200	Ab14917	Abcam (Cambridge, MA)
Podoplanin	Hamster monoclonal	1:500	Ab11936	Abcam
α-SMA	Mouse monoclonal Cy3-conjugated	1:1000	C6198	Sigma-Aldrich (Saint Louis, MO)
CD31	Rat monoclonal	1:200	553370	BD Biosciences (Franklin Lakes, NJ)
CD11b	Rat monoclonal	1:300	557395	BD Biosciences
VEGFR3/Flt-4	Goat polyclonal	1:100	AF743	R&D Systems
pAkt	Rabbit monoclonal	1:400	4060s	R&D Systems
CCL21	Goat polyclonal	1:40	AF457	R&D Systems
Cleaved Caspase-3	Rabbit monoclonal	1:100	MAB 835	R&D Systems
VE-Cadherin	Goat polyclonal	1:500	AF1002	R&D Systems
ICAM-1	Rat monoclonal	1:100	YN1/1.7.4 Ab119871	Abcam
p-Selectin	Rabbit monoclonal	1:500	EPR5047 Ab134047	Abcam
iNOS	Rabbit polyclonal	1:100	Ab3523	Abcam
CD3	Rabbit polyclonal	1:200	A0452	Dako (Santa Clara, CA)
Nitrotyrosine (N-Tyr)	Rabbit polyclonal	1:200	BS-8551R	BIOSS (Woodburn, MA)
Collagen I	Rabbit polyclonal	1:100	Ab34710	Abcam
MacroH2A1	Rabbit polyclonal	1:100	39593	Thermo Fisher
RYR1	Rabbit polyclonal	1:200	AB9078	Millipore

All sections were scanned using a Mirax slide scanner (Zeiss; Munich, Germany) and whole mounts were imaged using an SP-5 upright confocal microscope (Leica Microsystems; Wetzlar, Germany). Image analysis was performed with Pannoramic Viewer (3D Histech; Budapest, Hungary). Lymphatic vessel density (% total area), fluorescence intensity, mean fluorescence intensity and vessel diameter were measured using ImageJ software (NIH; available at https://imagej.nih.gov). Lymphatic vessel density and fluorescence intensity were analyzed using a ×20 magnification images (5 per mouse) and average values represented as graphs. Branch points and diameter were analyzed using ×20 magnification images (5 per mouse) by two blinded reviewers. To analyze peri-lymphatic inflammation cells or fluorescence intensity of structures within 100 μm radius of lymphatic vessels were counted by two blinded reviewers from five images per mouse and averages were plotted as graphs.

### Flow Cytometry and LEC Sorting

Single-cell suspensions of tissues (1 whole ear or 1 cm^2^ of back skin) were prepared by mechanical dissociation followed by incubation with digestion buffer containing collagenase D (0.2 mg/ml), DNAse I (0.1 mg/ml), and Dispase II (0.8 mg/ml) (Roche Diagnostics; Indianapolis, IN). Erythrocytes were lysed with RBC lysis buffer (eBioscience; San Diego, CA). Samples were stained with various combinations of the following fluorophore-conjugated mouse monoclonal antibodies: PE-anti-podoplanin (8.1.1; #127407), FITC-anti-CD45 (30-F11; #103107), PE-anti-CD11c (N418; #117307), APC-anti-CD31 (MEC13.3; #102509), APC-anti-CD11b (M1/70; #101211), BV-421-anti-Ly6C/6G (RB6-8C5; #108422), APC-anti-CD8a (53–6.7, #100712), and PE-CD45R/B220 (RA3-6B2, #103207) (all from Biolegend), APC-anti-CD3 (17A2; #17-0032-80), FITC or PE-anti-CD4 (GK 1.5; #11-0041-82, 12-0041-81) from eBioscience, PE-anti-LYVE-1 (cat # 12-0443-82) from Invitrogen. Non-specific staining was reduced with Fc receptor blocking (rat monoclonal anti-CD16/CD32; #14-0161-85; eBioscience). DAPI viability dye was also used to exclude dead cells. Single-stain compensation samples were created using UltraComp eBeads (#01-2222-42; Affymetrix, Inc.). Flow cytometry was performed using a BD Fortessa flow cytometry analyzer (BD Biosciences; San Jose, CA), and data were analyzed with FlowJo software (Tree Star; Ashland, OR). Percentage of LECs was quantified as %CD45 negative cells and LEC numbers were quantified as number of LECs per 100,000 cells. Similarly, immune cells were quantified as percentage of CD45+ cells or CD3+ cells and numbers were quantified as number of cells per 100,000 cells. LECs (CD45−, CD31+, podoplanin+ cells) were sorted from skin (following enzymic disassociation and staining of single-cell suspensions as described above) using a BD Aria 6 cell sorter. Cells were collected in Eppendorf tubes and processed immediately.

### Lymphatic Function Analysis

Lymphatic leakiness was assessed using FITC-conjugated lectin (#FL-1171, Vector Laboratories). Using an ultra-fine Hamilton syringe needle, 3 µg (1 μg/μl) of lectin was intradermally injected in the ear skin. After 5 min, ears were harvested and whole-mounted for immunofluorescent staining to identify the capillary and collecting lymphatic vessels draining the lectin.

Hindlimb collecting lymphatic vessel pumping was assessed using indocyanine green (ICG) near-infrared lymphangiography. After inducing anesthesia, 15 µl of ICG (0.15 mg/ml; Sigma-Aldrich) was intradermally injected into the first web space of the dorsal hindlimbs of each mouse. To promote ICG uptake into the lymphatic vasculature, mice were awakened and allowed to move freely for 30 min. Mice were then re-anesthetized for near-infrared imaging of the hindlimb using a custom-made EVOS EMCCD camera (Life Technologies; Carlsbad, CA) and LED light source (CoolLED; Andover, United Kingdom) mounted on a SteREO Lumar.v12 microscope (Zeiss; Jena, Germany). The same machine and settings were used to obtain images every 8 s for 30 min for each mouse hindlimb. Fiji software (National Institutes of Health; Bethesda, MD) was used to analyze lymphatic pumping. For uniformity, a region of interest was chosen over the dominant collecting vessel of each hindlimb. Near-infrared fluorescence intensity, which correlates with lymphatic contractions, was plotted over time as arbitrary units to subtract any noise. To avoid inaccuracies from inadvertent lymphatic stimulation due to positioning, the first 10 min of each image set were excluded.

### Dendritic Cell Migration Assay

Skin dendritic cell (DC) migration via lymphatic vessels was assessed using a modification of previously reported methods (Wendland et al., 2011). Briefly, 8% type I isomer fluorescein isothiocyanate (FITC; 5 mg/ml; Sigma Aldrich) was diluted in a 1:1 mixture of acetone and dibutylphthalate (Sigma Aldrich). A total of 10 µl of the solution was painted on each side of the mouse ear. The mice were then sacrificed at 20 h later to allow for collection of the draining cervical lymph nodes following ear painting. Single-cell suspensions were obtained from these lymph nodes by enzymatic digestion, from which FITC+CD11c+ DCs were analyzed by flow cytometry as previously described (Kataru et al., 2021).

### Enzyme-Linked Immunosorbent Assay and PCR

Protein isolated from lysates of sorted LECs from aged and young mouse skin was analyzed by ELISA to measure the concentration of pAkt (normalized to total Akt) using a commercially available ELISA kit (abcam-ab126433) following the manufacturer’s protocol. Each assay used 50 µg of homogenate and was performed in duplicate.

Gene expression in cell lysates of sorted skin LECs was measured by qPCR. RNA was isolated using RNeasy Micro kits (Qiagen). cDNA was generated using Maxima H Minus cDNA synthesis master mix and dsDNase (Thermo Fisher Scientific) using 8 µl of isolated RNA. qPCR was performed using Quanti Tect SYBR Green PCR master mix (Qiagen) using pre-validated primers for Prox1 (QT01070615), VEGFR3 (QT00102536), podoplanin (QT01552257), LYVE-1 (QT00158907), CCL21 (QT00284753), VE-cadherin (QT00110467), ZO-1 (QT00493899), ICAM-1 (QT00155078) and p-Selectin (QT00106379) (Qiagen, Hilden Germany) using 1 µg of cDNA/reaction on a ViiA 7 Real-time PCR system (Thermo Fisher) following the manufacturer’s protocol. After normalizing to GAPDH expression, mRNA expression was measured as fold change using the ΔΔCt method. Each qPCR assay was performed in triplicate.

### Statistical Analysis

Statistical analyses were performed using GraphPad Prism (GraphPad Software; San Diego, CA). Normal distribution of all data sets were checked using the Shapiro-Wilk normality test and unpaired Student’s *t*-test or the Mann-Whitney test were as appropriate. Data are presented as mean ± standard deviation unless otherwise noted, and *p* < 0.05 was considered significant.

## Results

### Aging Results in Decreased Skin Lymphatic Density

**FIGURE 1:**
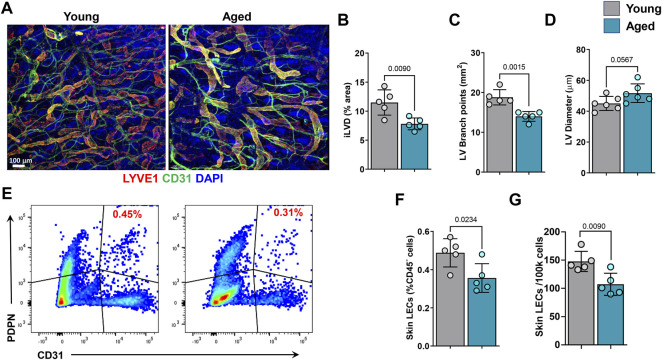
Aging causes decreases in initial lymphatic vessel density and lymphatic endothelial cell (LEC) numbers. **(A)** Representative confocal images of mouse skin showing initial lymphatic vessels (LYVE-1) and blood vessels (CD31) density. Quantification of initial lymphatic vessel **(B)** density, **(C)** branch points, and **(D)** vessel diameter. **(E)** Representative flow cytometry dot plots showing frequencies of CD45-/podoplanin (PDPN)+/CD31+ lymphatic endothelial cells. **(F)** Quantification of skin LECs as a percentage of CD45^−^ cells. **(G)** Quantification of the absolute number of skin LECs among 100,000 total cells. *n* = 5 mice per group for all experiments. All quantifications are mean ± SD, unpaired Student’s *t* test.

Consistent with a previous study, we found that aging is associated with decreased density and branching, by approximately 35 and 25%, respectively, of LYVE-1+ initial lymphatic vessels (iLV) (Figures 1A–C) (Karaman et al., 2015). In contrast, we found no effect of aging on iLV diameter (Figure 1D). To corroborate previous reports and our histological findings, we performed flow cytometry on ear skin tissue digests with gating to identify total LECs (CD45− CD31+ podoplanin+). This analysis also demonstrated that skin derived from aged mice contains fewer LECs relative to total CD45^−^ cells, as well as a 27% lower absolute number of total LECs compared to young mice (Figures 1E–G).

**FIGURE 2:**
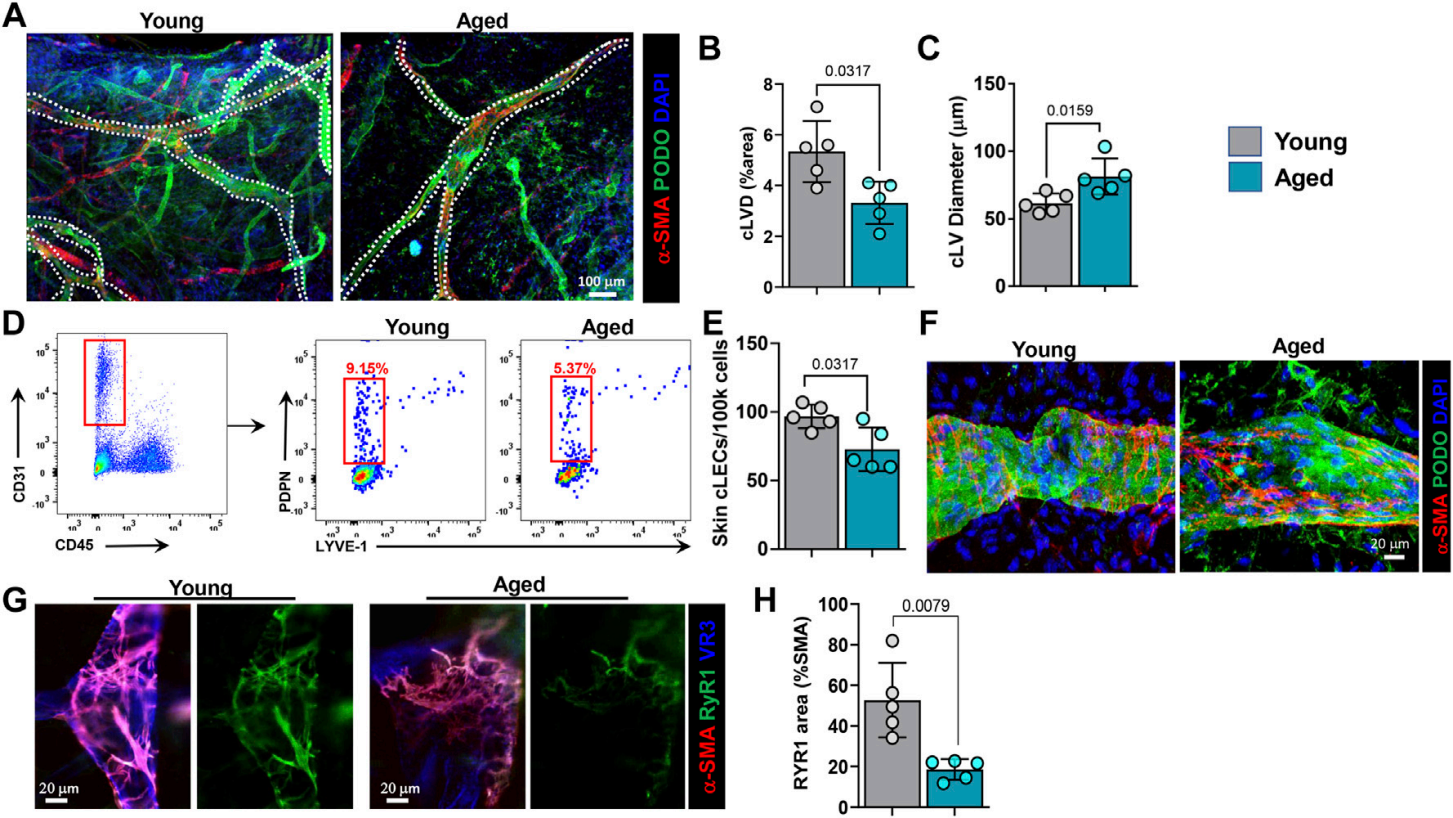
Aging causes decreases in collecting lymphatic vessel density and collecting LEC numbers and alters their phenotype. **(A)** Representative confocal images of mouse skin collecting lymphatic vessels showing colocalization of PDPN and α-SMA. Quantification of collecting lymphatic vessel **(B)** density and **(C)** diameter. **(D)** Representative flow cytometry dot plots showing frequencies of CD31+/CD45-/PDPN+/LYVE-1- collecting LECs. **(E)** Quantification of the absolute number of skin collecting vessel LECs among 100,000 total cells. **(F)** Representative high magnification confocal images of young and aged collecting lymphatic vessels showing orientation of LMCs (α-SMA) relative to LECs (PDPN). **(G)** Representative high magnification confocal images of young and aged collecting lymphatic vessels showing expression of ryanodine receptor 1 (RyR1) in LMCs (α-SMA). **(H)** Quantification of RyR1 density as a percentage of LMC density. *n* = 5 mice per group for all experiments. All quantifications are mean ± SD, unpaired Student’s *t* test.

We, next analyzed morphologic changes in collecting lymphatic vessels (cLVs) using ear whole mount sections stained for podoplanin (PDPN) and α-SMA (Figures 2A–C). This analysis showed that aged animals have fewer cLVs (38% decrease in total area) and that those remaining are significantly dilated (23% increase in diameter). We next used flow cytometry of ear skin digests to quantify collecting LECs (CD45−/CD31+/podoplanin+/LYVE-1-). This analysis confirmed our histological findings and showed that aging results in a decrease in collecting LECs as a percentage of both CD45− and total cells, by approximately 41 and 25%, respectively (Figures 2D,E). Further, high magnification confocal imaging of skin cLVs revealed abnormal LMC coverage in aged mice (Figure 2F). In cLVs from young mice, α-SMA+ LMCs were oriented perpendicular to LECs; in contrast, in cLVs from aged mice, LMCs were more parallel to LECs. This altered orientation of LMCs in aged cLVs likely affects their pumping capacity. Because ryanodine receptors (RyR, calcium-gated channels) play an important role in LMC contraction and expansion (Jo et al., 2019), we assessed their spatial distribution by high magnification confocal imaging. We observed a marked decrease in expression of RyR1 on the LMCs of aged mice compared to young mice (approximately 65% decrease) (Figures 2G,H).

### Aging Results in Decreased Dendritic Cell Migration and Impaired Lymphatic Pumping

**FIGURE 3:**
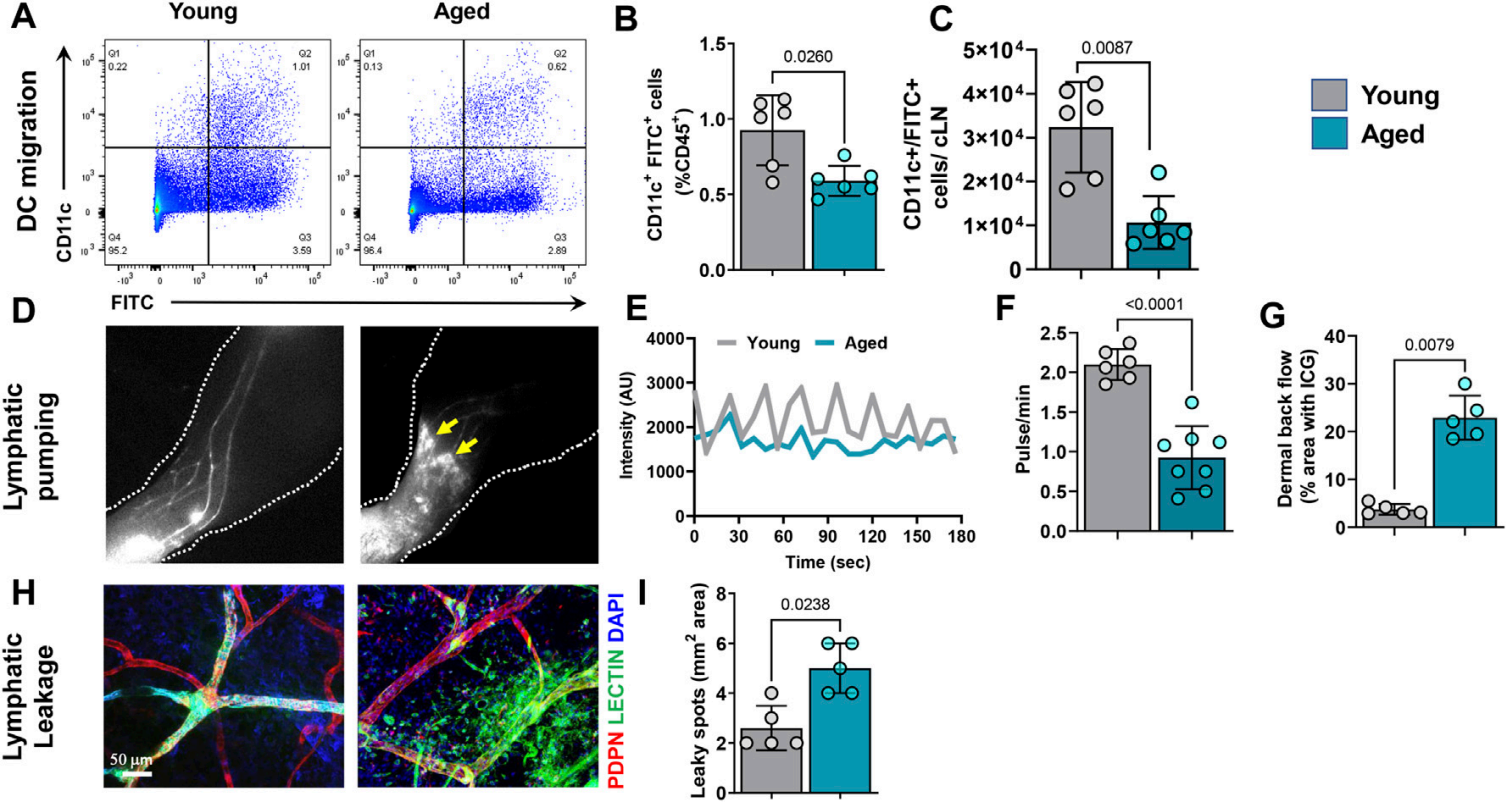
Aging causes decreases in dendritic cell (DC) migration and lymphatic pumping and increased leakiness in lymphatic vessels. **(A)** Representative dot plots of FITC+/CD11c+ DCs from deep cervical lymph nodes (which drain ear skin) after FITC painting of the ear skin. **(B)** Quantification of FITC+ DCs as a percentage of CD45+ cells from cervical lymph nodes. **(C)** Quantification total number of FITC+ DCs per lymph node. *n* = 6 mice per group from two independent experiments. **(D)** Representative hind limb ICG lymphangiography NIR images showing hind limb collecting lymphatic vessels, dermal backflow, and dye pooling (arrows) in aged mice. **(E)** Graphical representation of ICG intensity peaks plotted against time in hind limbs of young and aged mice. **(F)** Quantification of the number of lymphatic pulses per minute in young and aged mice. **(G)** Quantification of dye pooling, which represents dermal backflow. *n* = 5–7 mice per group from two independent experiments. **(H)** Representative confocal images of whole-mounted ear skin from young and aged mice showing FITC-lectin leakage in lymphatic vessels. **(I)** Quantification of FITC-lectin leakage (total FITC-lectin-containing area). *n* = 5 mice per group. All quantifications are mean ± SD, unpaired Student’s *t* test.

The effects of aging on lymphatic transport of antigen-presenting cells from the skin to draining lymph nodes is unknown. This is important because antigen presentation in the lymph node is necessary for immune responses. We therefore analyzed dendritic cell (DC) migration using an established assay in which fluorescein isothiocyanate (FITC) is painted onto the ear skin to label DCs. This analysis showed that migration of DCs (CD45+/CD11chigh/FITC+) to the draining cervical lymph nodes 20 h after FITC application was significantly impaired. We noted a decrease of 40% and 3-fold in FITC+/CD11c+ cells percentage and total numbers, respectively in aged animals (Figures 3A–C).

Next, we analyzed pumping function of skin cLVs in the hindlimb using ICG NIR lymphography. Time-lapse analysis of ICG-containing cLVs showed that aging results in significant reductions in both the frequency and amplitude of lymphatic contractions (Figures 3D,E and Supplementary Video S1). cLVs in young mice displayed a strong pulsatile function with regular high intensity peaks at any given region of interest. In contrast, aged cLVs showed irregular, low-intensity peaks. Quantification of intensity peaks, corresponding to lymphatic pumping (Sharma et al., 2007; Quan Zhou et al., 2010), revealed a marked reduction (57%) in the number of lymphatic pulsations in aged mice (Figure 3F). In addition, cLVs of aged mice also displayed areas of ICG accumulation in the skin consistent with lymphatic leakiness and dermal backflow (Figure 3G). To corroborate this finding, we visualized lymphatic vessel leakiness using confocal imaging of the ear skin injected with FITC-labeled lectin, a high molecular weight molecule that is exclusively transported through lymphatic vessels when injected intradermally (Figures 3H,I). In aged mice, marked extravasation of FITC-lectin from cLVs was observed, resulting in an approximately 50% increase in FITC-positive area. In contrast, FITC-lectin did not leak from the cLVs of young mice.

### Aging Results in Decreased LEC VEGFR3 Signaling

**FIGURE 4:**
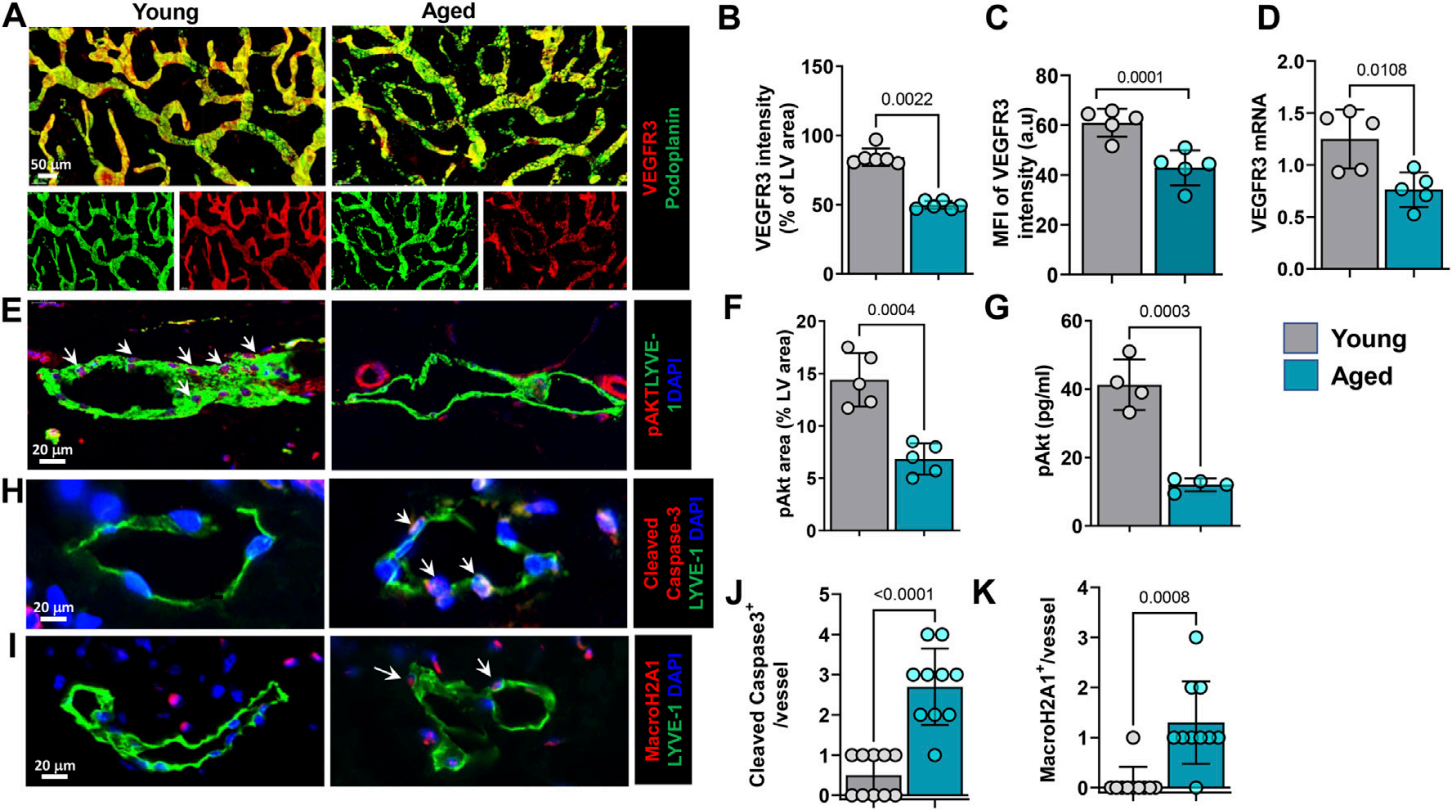
Aging causes decreased LEC VEGFR3 signaling and increased senescence and apoptosis. **(A)**. Representative confocal images of whole-mounted ear skin stained for podoplanin (PDPN) and VEGFR3. *n* = 6 mice per group. **(B)** Quantification of VEGFR3 immunostaining intensity. **(C)** Quantification of VEGFR3 mean fluorescence intensity (MFI). **(D)** Quantification of VEGFR3 mRNA expression in sorted skin LECs measured by qRT-PCR. *n* = 5 mice per group. **(E)** Representative confocal images of ear skin sections showing colocalization of lymphatics (LYVE-1) and pAkt. Arrows in young mouse skin indicate strong expression and colocalization of pAkt with LECs. **(F)** Quantification of pAkt immunostaining as a percentage of lymphatic vessel area. *n* = 5 mice per group. **(G)** ELISA quantification of pAkt (normalized to Akt) from lysates of sorted skin LECs. *n* = 4 mice per group. **(H)** Representative confocal images of ear skin sections stained for lymphatics (LYVE-1) and apoptosis marker cleaved caspase-3. Arrows highlight cleaved caspase-3 expression in aged mice LECs. **(I)** Representative confocal images of ear skin sections stained for lymphatics (LYVE-1) and senescence marker MacroH2A1. Arrows highlight MacroH2A1 expression in aged mice LECs. **(J,K)** Quantification of cleaved caspase-3+ **(J)** and MacroH2A1+ **(K)** LECs on immunostaining. *n* = 5 mice per group. All quantifications are mean ± SD, unpaired Student’s *t* test.

Because aging was associated with decreased lymphatic vessel density and branching, we next sought to determine whether these effects might be mediated in part by changes in VEGFR3 expression and activation. This pathway plays a critical role in regulating LEC proliferation, differentiation, and protection from apoptosis (Coso et al., 2014). Immunostaining of whole-mounted ear skin samples showed that aging was associated with an approximately 40% decrease in VEGFR3 immunoreactivity on lymphatic vessels (Figures 4A–C). This finding was corroborated by quantitative RT-PCR performed on sorted skin LECs, which demonstrated a similar decrease in VEGFR3 mRNA expression in LECs isolated from aged animals (Figure 4D). We also found that aging decreased the expression of the LEC-specific transcription factor Prox-1 by 57%. However, we noted no changes in the expression of LYVE-1 by immunostaining and RT-PCR or podoplanin by RT-PCR (Supplementary Figures S1A,B). VEGFR3 activation results in phosphorylation of Akt and is essential for lymphatic growth and proliferation (Deng et al., 2015). High magnification images of ear skin sections stained for pAkt showed that aged LECs had decreased intracellular pAkt compared with young mice (Figures 4E,F). This finding was corroborated using an ELISA to quantify pAkt in sorted LECs isolated from young and aged mouse skin (Figure 4G). This analysis showed that aging resulted in a more than 2-fold decrease in pAkt in LECs. VEGFR3 signaling and Akt phosphorylation in LECs provide protection from apoptosis and cellular senescence (Karaman et al., 2018). Consistent with our finding that aging decreases VEGFR3 expression and intracellular pAkt, we found that aging also resulted in increased expression of cleaved caspase-3, a marker of apoptosis, and histone macro H2A1 (mH2A1), a marker of senescence, by approximately 8- and 6-fold, respectively (Figures 4H–K).

### Lymphatics in Aging Animals Are Surrounded by Leukocytes and Nitrosative Stress Conditions

Low-grade inflammation is a pathologic characteristic of aging and many other chronic conditions (Frasca et al., 2017). Previous reports have shown that aging leads to increased mast cell infiltration around lymphatic vessels (Pal et al., 2017). Therefore, we sought to determine if aged mice have increased skin infiltration of inflammatory cells. Flow cytometry analysis of mouse skin showed that the percentage and number of CD45+ leukocytes was significantly decreased in aged skin by approximately 30 and 35%, respectively. Despite fewer total CD45+ cells in aged tissues, the percentage and number of myeloid cells (neutrophils and macrophages) was significantly increased in skin harvested from aged mice. The percentage of neutrophils in aged tissue was increased by 5-fold and the total number by 4.5-fold. The percentage of macrophages increased by 5-fold and total number by 3-fold. The percentage and number of DCs was not altered by aging. We found no differences in the percentage of CD3+ T cells, however, the absolute number of T cells decreased in aging skin by approximately 40%. We also found no differences in the percentage of T helper cells, but the absolute number decreased by approximately 45%; in contrast, the percentage, and the absolute number of cytotoxic T cells was significantly increased (approximately 2.5-fold and 2-fold respectively) in aging mice. We also found a more than 3-fold decrease in percentage of B220+ B cells and 4-fold decrease in total number of B cells in aged skin compared to young skin (Supplementary Figures 2A–C).

**FIGURE 5:**
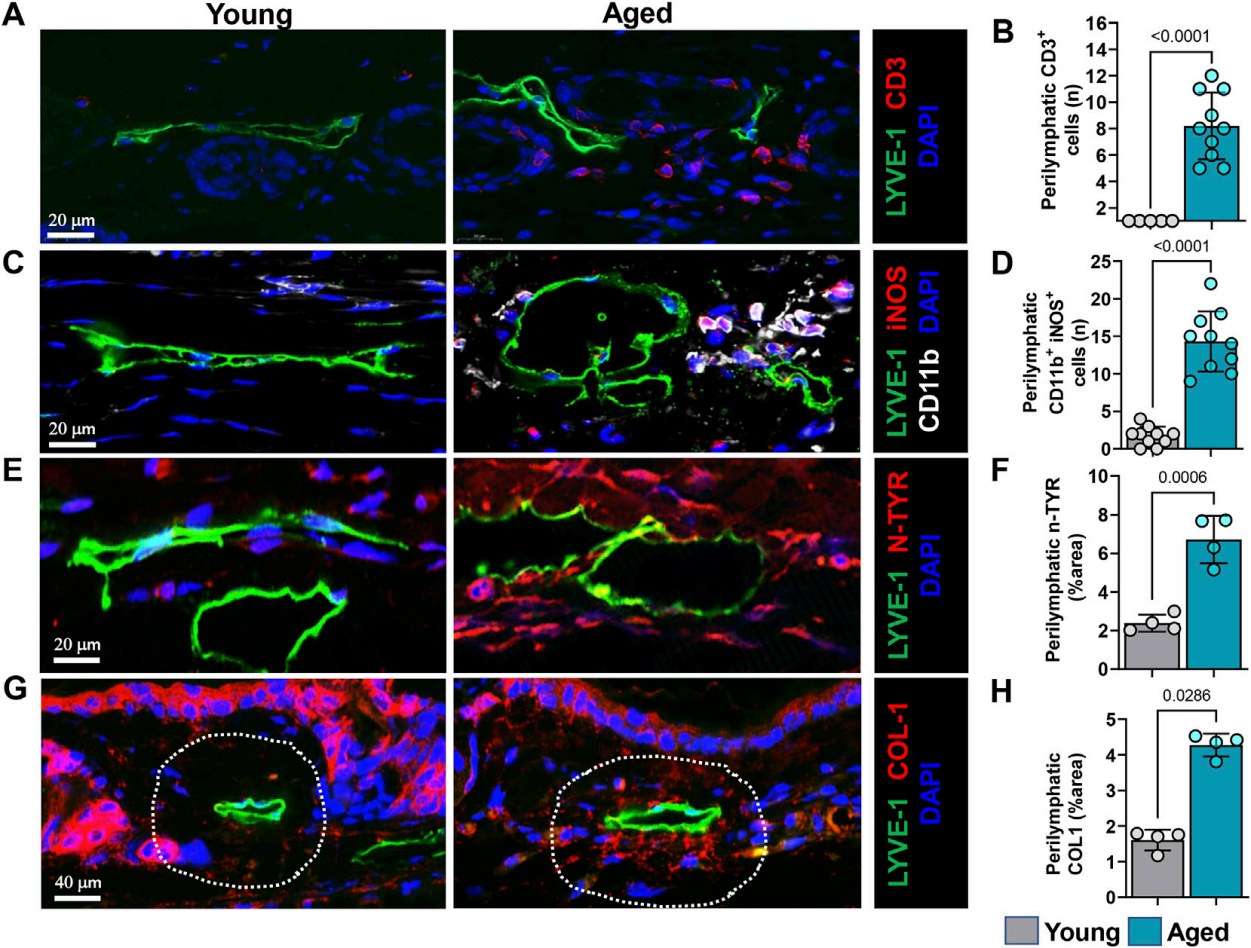
Aging causes increased immune cell infiltration and nitrosative stress surrounding lymphatics. **(A)** Representative confocal images of ear skin sections stained for lymphatic vessels (LYVE-1) and T cells (CD3). **(B)** Quantification of T cells within 100 µm radius of lymphatic vessels. **(C)** Representative confocal images of ear skin sections stained for lymphatic vessels (LYVE-1) and myeloid cells (CD11b+/iNOS+). **(D)** Quantification of myeloid cells within 100 µm radius of lymphatic vessels. **(E)** Representative confocal images of ear skin sections stained for lymphatic vessels (LYVE-1) and nitrotyrosine (N-Tyr), a marker of nitrosative stress. **(F)** Quantification of N-Tyr positivity within 100 µm radius of lymphatic vessels. **(G)** Representative confocal images of ear skin sections stained for lymphatic vessels (LYVE-1) and collagen-1. **(H)** Quantification of collagen-1 deposits within 100 µm radius of lymphatic vessels. *n* = 5 mice per group in all experiments. All quantifications are mean ± SD, unpaired Student’s *t* test.

To characterize the spatial distribution of these immune cells relative to lymphatic vessels we performed immunostaining in the tissue sections. Immunohistochemical staining of ear skin showed increased numbers of T cells (CD3+) and myeloid cells (CD11b+) around papillary and reticular dermis in aged but not young mice. Interestingly, these CD3+ inflammatory cells tended to cluster in a peri-lymphatic fashion, usually located within 100 µm of the lymphatic wall (Figures 5A,B).

Increased expression of inducible nitric oxide synthase (iNOS) by inflammatory cells could cause lymphatic dysfunction. in similar lines, co-localization of CD11b with iNOS antibodies demonstrated that, expression of iNOS by CD11b+ cells in aged mice but not in young. These CD11b+/iNOS+ cells are located predominantly adjacent to lymphatics, like CD3 T cells (Figures 5C,D and Supplementary Figures 3A). High levels of iNOS expression increase tissue nitric oxide (NO) concentrations and generate reactive nitrogen and oxygen species. LECs are highly sensitive to reactive nitrogen/oxygen species and exposure to even small amounts decreases cellular VEGFR3 expression, decreases cellular proliferation, and can induce apoptosis (Rehal et al., 2020). To analyze accumulation of reactive nitrogen species around lymphatic vessels, we evaluated the co-localization of lymphatic vessels with nitrotyrosine because tyrosine nitration is a post-translational modification caused by reactive nitrogen species. This analysis demonstrated extensive tyrosine nitration in the tissues immediately surrounding LYVE-1+lymphatic vessels in the skin of aged but not young mice, corresponding to an approximately 3-fold increase (Figures 5E,F and Supplementary Figure S3B). Diminution of dermal collagen is also a hallmark of aged skin. However, interestingly, when we investigated collagen arrangement near lymphatic vessels, we observed accumulation of collagen bundles near lymphatic channels in aged mouse skin (approximately 2.5-fold more than in young mice), despite decreased epidermal and dermal type I collagen (Figures 5G,H), confirming prior studies (Russell-Goldman and Murphy, 2020). This increased peri-lymphatic collagen deposition may result from chronic inflammation, which regulates collagen deposition and impairs parenchymal functioning in fibroproliferative disorders (Ueha et al., 2012).

Accumulation of antibody deposits in peripheral tissues is a hallmark of age induced autoimmune diseases and it is reported that lymphatic function regulates antibody deposits in the skin (Thomas et al., 2012). Therefore, we looked for antibody deposits in the skin of aged mice by immunostaining. Our results showed approximately 6-fold greater peri-lymphatic accumulation of IgG antibodies around lymphatic vessels in the skin of aged but not young mice (Supplementary Figures S3A,B).

### Aging Alters Chemokine and Junctional Protein Expression by LECs

**FIGURE 6:**
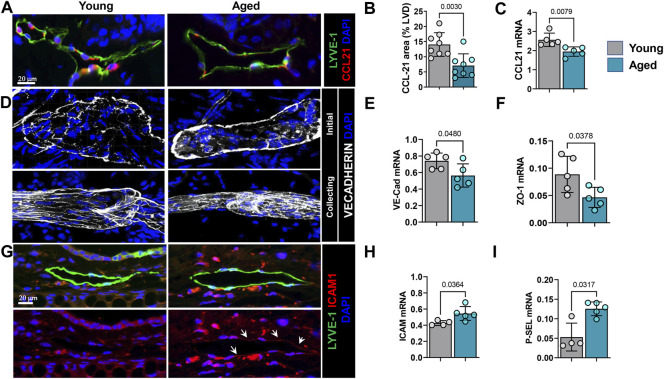
Aging causes decreased chemokine and altered junctional protein expression by LECs. **(A)** Representative confocal images of ear skin sections stained for lymphatic vessels (LYVE-1) and CCL21. **(B)** Quantification of CCL21 immunostaining as a percentage of lymphatic vessel area. **(C)** qRT-PCR quantification of CCL21 mRNA expression in sorted skin LECs. **(D)** Representative confocal images of ear skin wholemount stained for nuclei (DAPI), VE-cadherin (upper panel, Initial lymphatics; lower, collecting lymphatics). **(E)** qRT-PCR quantification of VE-cadherin and **(F)** ZO-1 mRNA expression in sorted skin LECs. **(G)** Representative confocal images of ear skin sections stained for lymphatic vessels (LYVE-1) and ICAM-1 (upper panel, immunofluorescence of both; lower, ICAM-1 only). **(H)** qRT-PCR quantification of mRNA expression of endothelial adhesion molecules ICAM-1 and **(I)** p-Selectin from sorted skin LECs. *n* = 5 mice from each group. All quantifications are mean ± SD, unpaired Student’s *t* test.

Lymphatic vessels are a major route of inflammatory cell exit from the skin (Hunter et al., 2016; Hampton and Chtanova, 2019). Entry of inflammatory cells into lymphatic channels is regulated by interactions between CCR7, a cell surface receptor on inflammatory cells, and its ligand, CCL21. Lymphatic endothelium is a major source of CCL21 and gradients of this cytokine guide inflammatory cells to enter lymphatic channels. Immunohistochemical localization of CCL21 in ear skin demonstrated that aging results in an approximately 2-fold decrease in CCL21 expression by LECs (Figures 6A,B). This observation was corroborated by quantitative RT-PCR of sorted LECs which showed downregulation of CCL21 expression by approximately 25% in aged LECs (Figure 6C).

While CCL21 gradients serve as a chemoattractant for immune cells towards lymphatic vessels, the button-like VE-cadherin junctions between LECs facilitate immune cell entry into the lymphatic capillary lumen (Stritt et al., 2021). Wholemount immunofluorescence analysis of ear skin samples showed that capillary lymphatic vessels in young mice had the typical button-like pattern of VE-cadherin expression and collectors showed continuous zipper-like pattern (Figure 6D). In contrast, lymphatic capillaries in aged mice skin showed a disorganized and continuous VE-cadherin junction around the perimeter of the vessel and collectors showed rugged and partially discontinuous junctions (Figure 6D). Quantitative RT-PCR analysis of sorted LECs showed a significant reduction in VE-cadherin (approximately 25%) and ZO-1 (approximately 2-fold) expression in LECs from aged skin compared to those isolated from young mice (Figure 6E).

ICAM-1 is a transmembrane protein that plays an important role in leukocyte-endothelial transmigration (Rahman and Fazal, 2009). Consistent with our observation that aging results in peri-lymphatic accumulation of inflammatory cells, we noted that expression of ICAM-1 was significantly increased in ear skin capillary lymphatic LECs (Figure 6G). Our qRT-PCR data from sorted LECs also showed upregulation of ICAM-1 and p-Selectin, another endothelial surface molecule that plays an important role in binding and recruitment of inflammatory cells to tissues, by approximately 25 and 60%, respectively (Figures 6H,I). Taken together, our studies show that aging results in chronic, low-grade inflammation and that inflammatory cells tend to localize around lymphatic vessels due to decreased lymphatic function and changes in lymphatic cytokine and cell surface receptor expression.

## Discussion

### Aging Decreases Lymphatic Vessel Density

In this study, we have shown that aging markedly decreases the density of both capillary and collecting lymphatic vessels and causes morphologic changes in LMCs. We showed that these changes correlate with impaired lymphatic vessel pumping, leakiness of capillary and collecting lymphatics, increased expression of apoptotic and senescence markers, and impaired trafficking of DCs from the periphery to draining lymph nodes. Our findings are consistent with, and add to, previous studies demonstrating a decreased capillary lymphatic vessel density in aged mice (Karaman et al., 2015). Importantly, we corroborated our findings using flow cytometry to show that aging has negative consequences on both initial and collecting lymphatics.

### Aging Results in Impaired cLV Pumping

Previous studies on mesenteric lymphatics *in vivo* and *in vitro* have shown that aging significantly impairs lymphatic pumping and is associated with decreased expression of contractile proteins and decreased pumping efficiency (Gashev and Zawieja, 2010; Gashev and Chatterjee, 2013). Consistent with this, using ICG lymphangiography we found that aging also decreases skin collecting lymphatic pumping and is associated with leakiness in these vessels. Because ICG lymphangiography is commonly used clinically to quantify lymphatic pumping and functional capacity, these results are likely clinically relevant (Boni et al., 2015; Jørgensen et al., 2021). Anatomically, we found that the LMC morphology is altered by aging, resulting in a more longitudinal orientation instead of the circular orientation observed in young rodents. This anatomic change may decrease pumping efficiency or cause vessel dilation, thus promoting lymphatic dysfunction (Bridenbaugh et al., 2013; Razavi et al., 2020). Moreover, we observed that aging markedly decreased LMC RyR1 expression. Because RyR1 is responsible for release of Ca2+ from the LMCs, coordinating rhythmic contractions (Lanner et al., 2010; Jo et al., 2019), this finding may provide a mechanistic rationale for the impaired pumping observed in aging lymphatics that could be investigated in future studies.

### Aging Decreases LEC VEGFR3 Expression and Intracellular Akt Phosphorylation

Although numerous studies have shown that aging results in functional deficits in the lymphatic system, the cellular mechanisms that regulate these changes remain unknown and have not been explored (Shang et al., 2019). In the current study, we show that aging causes a significant downregulation of LEC VEGFR3 expression and is associated with decreased concentrations of LEC intracellular pAkt. This finding suggests that aged LECs may have a decreased sensitivity to VEGFC stimulation because activation of VEGFR3 by this growth factor promotes LEC proliferation, differentiation, and migration, and protects LECs from apoptosis and senescence via Akt phosphorylation (Mäkinen et al., 2001; Fei Zhou et al., 2010). Indeed, transgenic mice that lack VEGFC or VEGFR3 fail to develop a lymphatic system and die *in utero* (Dumont et al., 1998; Karkkainen et al., 2004). Even haplo-deficiency of either VEGFC or VEGFR3 causes lymphatic insufficiency in mice (Dellinger et al., 2007). Consistent with these previous studies, we found that aging resulted in increased expression of apoptosis and senescence markers. We have noted similar effects on LEC VEGFR3/intracellular pAkt expression in other pathological conditions such as obesity, in which lymphatic function is impaired, suggesting that this mechanism may be a common means by which lymphatic vessels become dysfunctional (García Nores et al., 2016). Furthermore, this hypothesis is also supported by previous studies demonstrating that age-related impairment in PI3K/Akt signaling is also associated with blood endothelial dysfunction (Smith and Hagen, 2003; Trott et al., 2013). Indeed, abnormalities in PI3K signaling have been implicated in age-related defects in cellular regeneration in other tissues, suggesting that this may be a generalized conserved cellular response to aging (Chen et al., 2013). This is an interesting area of research and requires significant additional study.

### Aging Results in Peri-Lymphatic Accumulation of Inflammatory Cells

Immune cell migration is an important function of the lymphatic system and is dependent on the lymphatic channels’ structural integrity and LEC expression of chemokines and adhesion molecules (Hampton and Chtanova, 2019; Arasa et al., 2021). We found that aging markedly decreased trafficking of DCs from the periphery to the draining LN. This finding is consistent with other pathological conditions, such as obesity and lymphedema, in which lymphatic function is impaired (Weitman et al., 2013; García Nores et al., 2018). In addition, decreased DC trafficking may contribute to age-related deficiencies in immune responses (Ponnappan and Ponnappan, 2011). Consistent with previous reports, we found that aging resulted in a low-grade chronic inflammatory response in the skin (Zhuang and Lyga, 2014). More intriguingly, we found that most inflammatory cells in skin were clustered around lymphatic vessels. LECs in aging mouse skin had decreased expression of CCL21 that can cause decreased immune cell chemotaxis into lymphatic vessels. Interestingly, LECs in aging skin upregulated expression of ICAM-1 and P-selectin. It is well known that acute inflammation upregulates ICAM-1 expression on lymphatic vessels and increases leukocyte transmigration (Johnson et al., 2006). On the contrary, increased expression of adhesion molecules can cause immune cell arrest and accumulation on endothelium, especially in the absence of flow causing formation of tertiary lymphoid structures (Figenschau et al., 2018). Considering the decreased lymphatic function and pumping downstream, it will be interesting to know whether increased ICAM-1 on aged lymphatics helps leukocyte migration or arrest. Aging LECs also had altered spatial expression patterns of junctional proteins that may act as a barrier to leukocyte entry. Taken together, these findings suggest that changes in LEC gene expression may promote accumulation of inflammatory cells in the skin and that loss of gradients of chemokines such as CCL21 in combination with changes in the distribution of cell surface molecules that regulate leukocyte-endothelial cell interactions underlie this phenotype. Alternatively, it is possible that decreased DC trafficking and skin inflammation may be related to the decreased lymphatic vessel density noted in aged animals. However, this hypothesis is challenged by previous reports demonstrating normal DC migration even in animals with severe lymphatic hypoplasia (Platt et al., 2013).

Tissue deposition of antibodies is a hallmark of autoimmunity, especially in aging (Vadasz et al., 2013). Previous studies using K14-VEGFR3-Ig mice showed that lymphatic deficiency causes accumulation of autoantibodies in the skin (Thomas et al., 2012). Accordingly, we noted increased antibody deposits in the skin of aged mice, especially in a peri-lymphatic fashion, and we speculate that decreased lymphatic function might contribute to this antibody deposition.

Inflammatory cell accumulation around LECs may contribute to age-related lymphatic dysfunction. We found that most peri-lymphatic myeloid cells in aged mice expressed iNOS. This was associated with extensive peri-lymphatic tyrosine nitration, suggesting that the concentration of reactive nitrogen species (RNS) (and most likely reactive oxygen) is increased in and around the lymphatics of aged mice. Using a diet-induced model of obesity, we have previously shown that LECs are highly sensitive to injury by reactive nitrogen species (Rehal et al., 2020). Exposure of LECs to even low doses of RNS markedly decreases LEC proliferation; higher doses induce apoptosis. More importantly, RNS in obesity regulate expression of VEGFR3 and Prox-1, suggesting that similar mechanisms may be responsible for our observation that aging decreases the expression of these molecules in LECs. Interestingly, in our previous study we showed that RNS decrease insulin sensitivity. Because the insulin receptor—like VEGFR3—is a tyrosine kinase, it is possible that exposure of LECs to RNS not only decreases expression of VEGFR3 but also decreases responsiveness to ligand binding. This hypothesis is intriguing but obviously requires additional research.

Our study has some limitations that we acknowledge. The primary limitation of our work is its observational nature. Our study also lacks specific manipulation of lymphatic vessels thus preventing us from attributing the skin inflammation we noted in aging animals exclusively to lymphatic dysfunction. Indeed, other mechanisms may be involved. For example, aging is associated with extravasation of inflammatory cells into the tissues owing to vascular hyperpermeability (Oakley and Tharakan, 2014). However, it is well known that lymphatic injury or genetic/pharmacological inhibition of VEGF-C/VEGFR3 signaling results in the accumulation of inflammatory cells in the tissues (Kataru et al., 2019; Schwager and Detmar, 2019). Thus, lymphatic vessels can regulate tissue inflammation during aging directly or indirectly due to poor clearance. Further, detailed lymphatic intervention studies are needed to understand this scenario of inflammation and lymphatic dysfunction during aging. However, we feel that our results are intriguing and are hopeful that these findings will provide hypotheses to be tested in further research. Nevertheless, with this limitation in mind, we conclude that aging results in significant alterations in lymphatic function in mouse skin. Chronic inflammatory responses and changes in LEC gene expression may underlie these functional deficits and require additional study.

## Data Availability

The original contributions presented in the study are included in the article/Supplementary Material, further inquiries can be directed to the corresponding author.

## Supplementary Material

Supplementary Figure S1
**(A)** Quantification of LYVE-1 fluorescence intensity. **(B)** qRT-PCR quantification of mRNA expression of lymphatic markers LYVE-1 and PDPN and Prox-1 from sorted skin LECs. *n* = 5 mice from each group. All quantifications are mean ± SD, unpaired Student’s *t* test.

Supplementary Figure S2 Flow cytometry quantification of skin immune cells including neutrophils, macrophages DCs, T lymphocytes (T helper and cytotoxic) and B cells in mouse skin. **(A)** Schematic experimental design on how immune cells were quantified by flow cytometry. **(B)** Quantification of the percentage of immune cells. **(C)** Quantification of the total number of immune cells per 100K cells counted. *n* = 5 mice per group in all experiments. All quantifications are mean ± SD, unpaired Student’s *t* test.

Supplementary Figure S3 Peri-lymphatic of myeloid cells and nitrosative stress. **(A)** Representative low magnification images of young and aged skin stained for lymphatics (LYVE-1) and myeloid cells (CD11b). **(B)** low magnification images of peri-lymphatic nitrosative stress visualized by N-TYR immunostaining. *n* = 5 mice per group in all experiments.

Supplementary Figure S4 Aging causes increased peri-lymphatic antibody deposition. **(A)** Representative confocal images of ear skin sections stained for lymphatic vessels (LYVE-1) and IgG. **(B)** Quantification of antibody deposits within 100 µm radius of lymphatic vessels as a percentage of lymphatic vessel area. All quantifications are mean ± SD, unpaired Student’s *t* test.

Supplementary Video S1 Representative time lapse video of hindlimb collecting lymphatic vessel pumping in young mouse assessed using indocyanine green (ICG) near-infrared lymphangiography.

Supplementary Video S2 Representative time lapse video of hindlimb collecting lymphatic vessel pumping in aged mouse assessed using indocyanine green (ICG) near-infrared lymphangiography.
