# Supplementary figures and images for: Structural and Functional Changes in Aged Skin Lymphatic Vessels
Source: Front Aging. 2022 Apr 4;3:864860. doi: 10.3389/fragi.2022.864860 (PMC9261401; doi:10.3389/fragi.2022.864860)

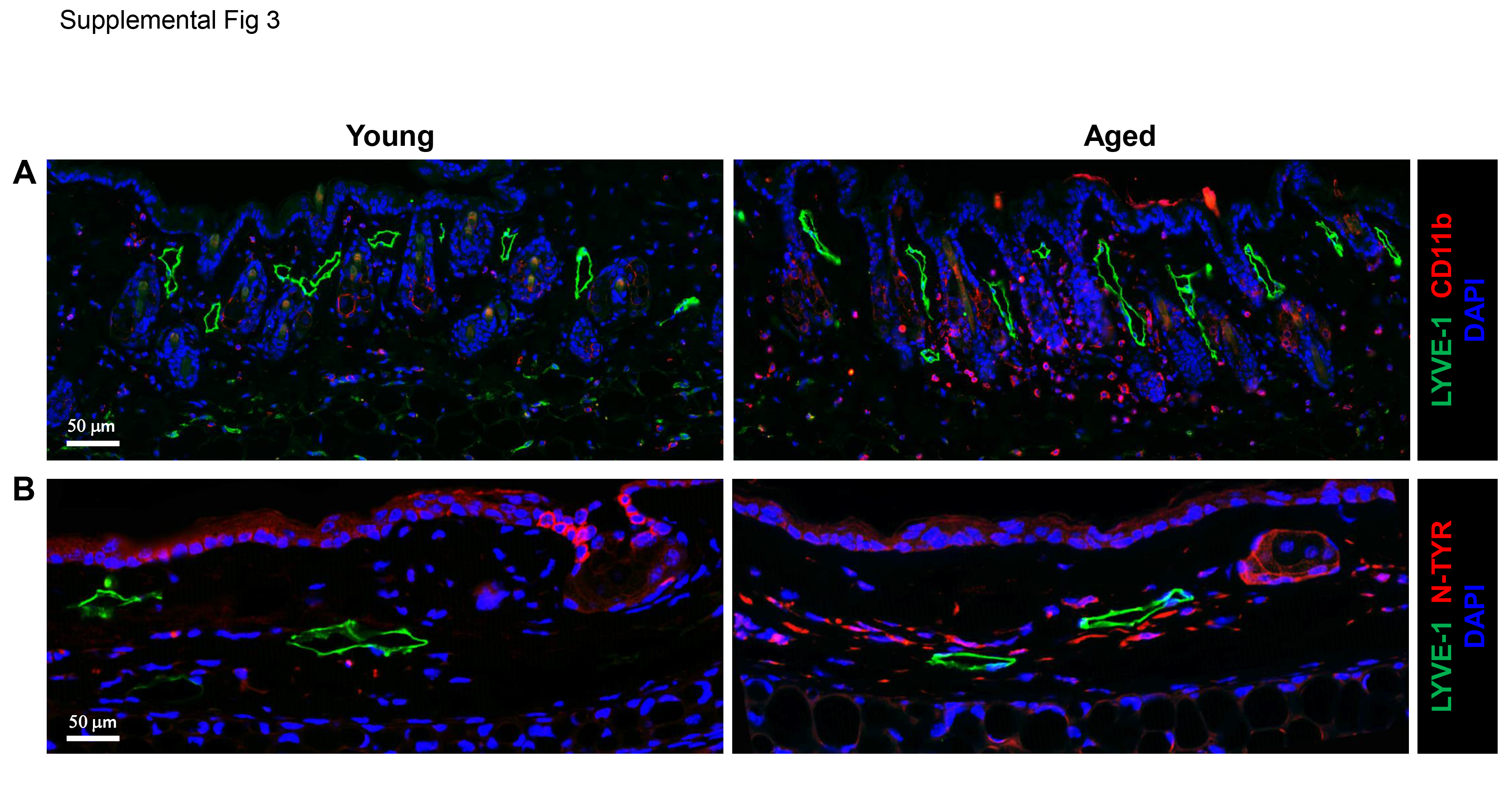

Supplement: Supplementary file 2 [file Image3.TIF]

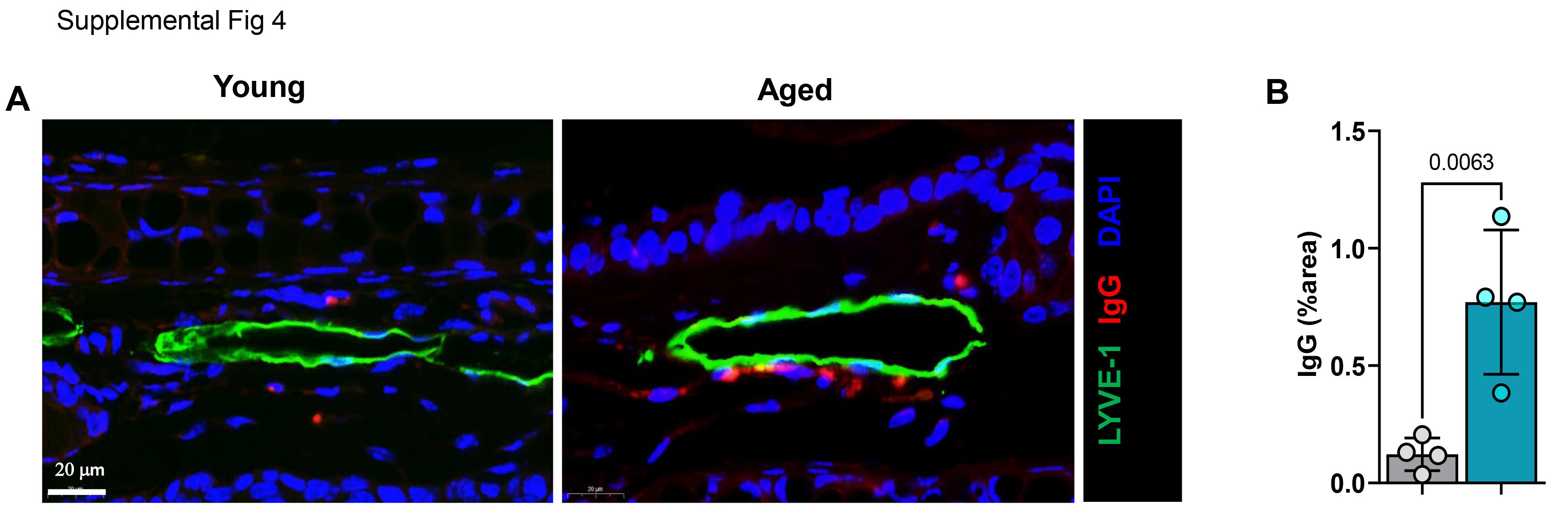

Supplement: Supplementary file 3 [file Image4.TIF]

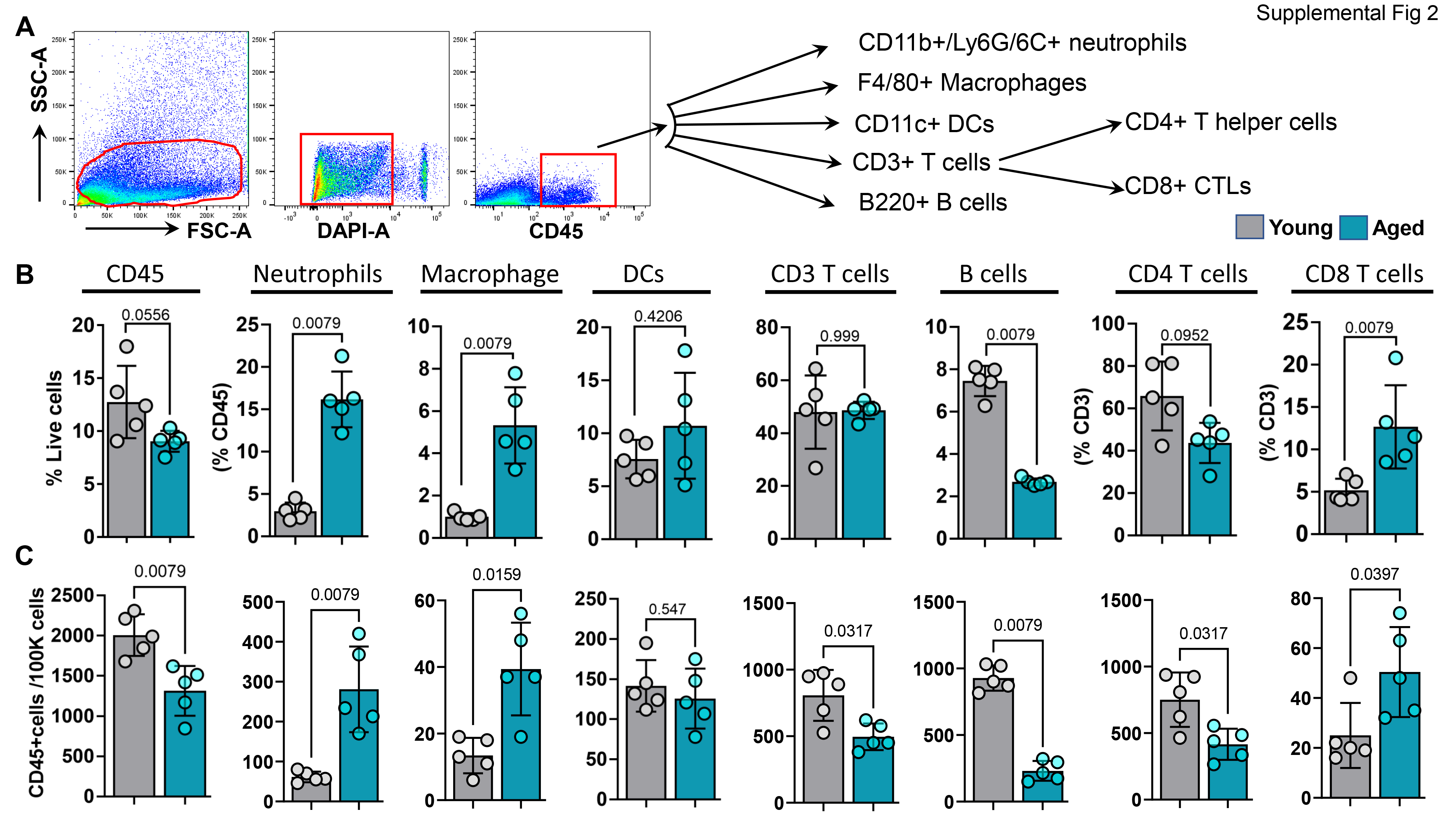

Supplement: Supplementary file 4 [file Image2.TIF]

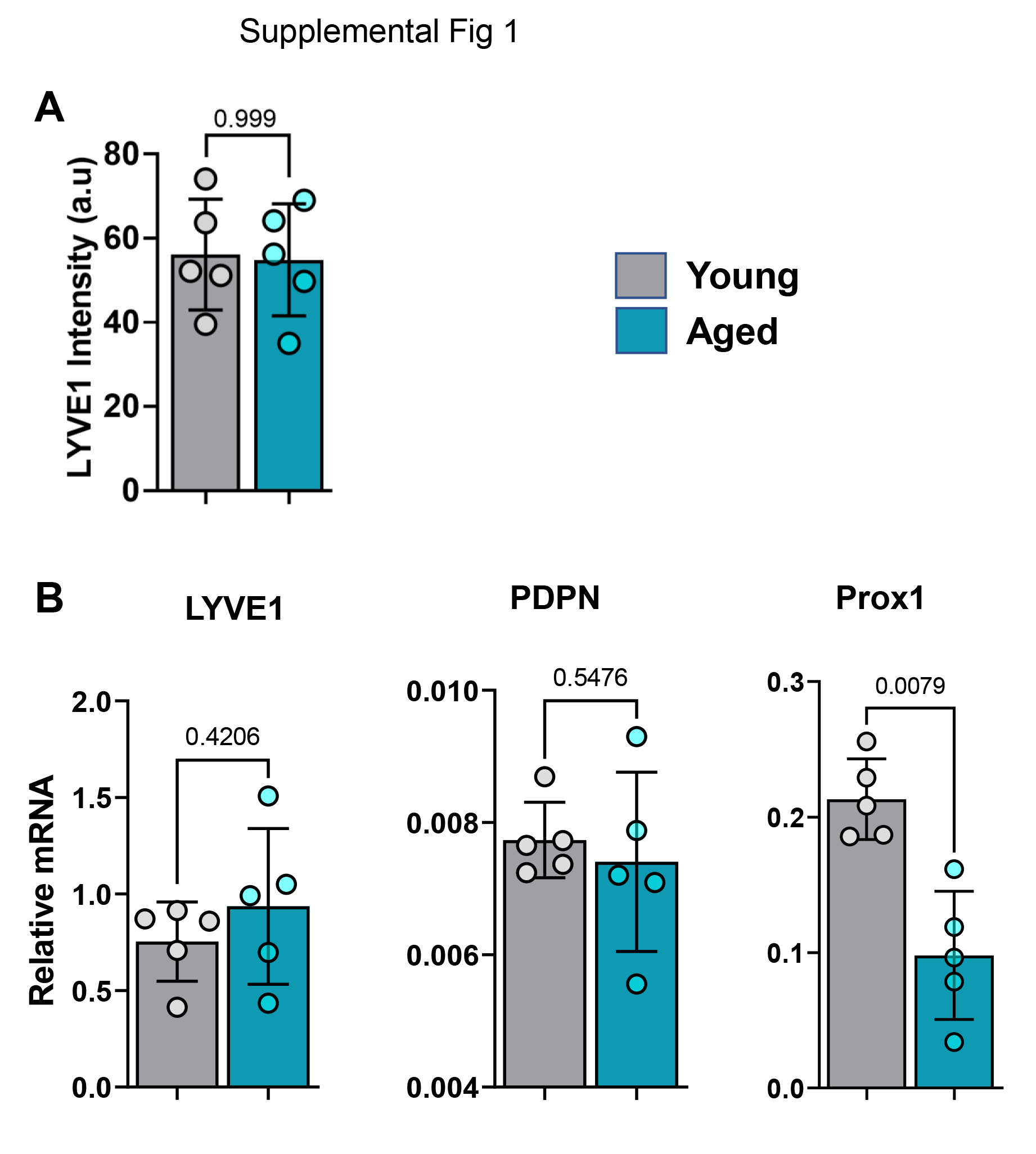

Supplement: Supplementary file 5 [file Image1.TIF]
